# Flatness of the Meckel cave may cause primary trigeminal neuralgia: a radiomics-based study

**DOI:** 10.1186/s10194-021-01317-4

**Published:** 2021-09-03

**Authors:** Jinzhi Lin, Yong Zhang, Wuming Li, Jianhao Yan, Yiquan Ke

**Affiliations:** 1 The National Key Clinical Specialty, The Engineering Technology Research Center of Education Ministry of China, Guangdong Provincial Key Laboratory on Brain Function Repair and Regeneration, Department of Neurosurgery, Zhujiang Hospital, Southern Medical University, 510282 Guangzhou, China; 2 Department of Neurosurgery, Guangdong Second Provincial General Hospital, 510317 Guangzhou, China

**Keywords:** flatness, Meckel cave, radiomics, trigeminal neuralgia, cause

## Abstract

**Background:**

Neurovascular contact (NVC) is the main cause of primary trigeminal neuralgia (PTN); however, cases of PTN without NVC are still observed. In this study, the Meckel cave (MC) morphology in PTN were analyzed by radiomics and compared to healthy controls (HCs) to explore the cause of PTN.

**Methods:**

We studied the 3.0T MRI data of 115 patients with PTN and 46 HCs. Bilateral MC was modeled using the 3D Slicer software, and the morphological characteristics of MC were analyzed using the radiomics method.

**Results:**

The right side incidence rate in the PTN group was higher than the left side incidence. By analyzing the flatness feature of MC, we observed that the affected side of the PTN was lower than that of the unaffected side, the right MC of the PTN and HC was lower than that of the left MC, the MC of the affected side of the left and right PTN without bilateral NVC was lower than that of the unaffected side.

**Conclusions:**

By providing a method to analyze the morphology of the MC, we found that there is an asymmetry in the morphology of bilateral MC in the PTN and HC groups. It can be inferred that the flatness of the MC may be a cause of PTN.

## Key points

Flatness of the Meckel cave may be a cause of primary trigeminal neuralgia.

There is asymmetry in the morphology of bilateral Meckel cave of primary trigeminal neuralgia and healthy controls.

The shape features of the Meckel cave can be accurately quantified and analyzed by radiomics method.

## Introduction

Trigeminal neuralgia (TN) is a chronic neuropathic pain disorder [1] that can lead to poor quality of life and even suicide [2]. TN can be classified as primary TN (PTN), which is either classical or idiopathic depending on the degree of neurovascular contact (NVC), or as secondary TN, which is caused by pathology rather than NVC [3]. Asymptomatic NVC in patients with PTN [4] and high prevalence of NVC in individuals without TN [5] suggest that NVC is not the only pathogeny of PTN, which is worthy of further study.

Anatomically, the intracranial trigeminal nerve is divided into the root entry zone (REZ) and the Meckel cave (MC) segments after emerging from the brain stem. Many studies [1–3] have proven that morphological changes (atrophy or displacement) with NVC of the trigeminal nerve in the REZ are related to symptomatic PTN. However, no significant correlation has been observed between PTN and NVC without morphological changes. Some unknown etiological factors are likely to play an important role [6]. The MC, an important structure after the REZ of the trigeminal nerve, is hence worth studying.

Previous studies on the trigeminal nerve morphology of PTN have focused on the REZ and lacked emphasis on the MC. The MC is an important part of the trigeminal nerve, which is composed of numerous small fibers and the trigeminal ganglion consisting of a small amount of solid tissue [7]. Some cases studies [8, 9] have reported that ipsilateral hypoplastic MC, which may lead to morphological atrophy, as well as crowded MC, may produce symptoms consistent with TN. Percutaneous balloon compression (PBC) can effectively relieve the pain symptoms of patients with PTN by changing the shape of the MC, which expands after balloon dilation; moreover, the balloon shape is a parameter that has a very strong impact on outcome [10, 11]. Therefore, it can be inferred that the morphological characteristics of the MCs may be related to PTN.

Here, we hypothesized that MCs with narrow morphological characteristics are related to PTN. Magnetic resonance imaging (MRI) provides the best way to evaluate the morphological characteristics of PTN [6], and radiomics can automatically quantify the phenotypic characteristics of medical imaging [12]. The aim of the present study was to collect 3.0T MRI data of patients with PTN and healthy controls (HCs) to accurately model the bilateral MC with the 3D Slicer software and then analyze the MC morphology using radiomics to explore the cause of PTN.

## Materials

### Data source

The study was approved by the Medical Ethics Committee of the Guangdong Second Provincial General Hospital in Guangzhou, China. Written informed consent was obtained from each subject. From January 2018 to January 2020, 115 PTN patients (mean age: 62.89 ± 10.43 years, 70 female) were recruited from the Department of Neurosurgery of the Second Provincial General Hospital of Guangdong. The PTN diagnosis was based on the Third edition of International Classification of Headache Disorders [13]. Exclusion criteria were secondary TN, other primary headache disorders (e.g., migraine or tension-type headache) and past surgical treatment of PTN (such as microvascular decompression, PBC, and radiofrequency). We also recruited 46 healthy volunteers as HC (mean age: 49.28 ± 10.96 years, 31 female). Table 1 lists the demographics and patient characteristics.

**Table 1:** Demographic characteristics of PTN and HC group

	PTN	HC	Total	*P*
**Case number(n,%)**	115(71.4)	46(28.6)	161	
**MC number(n,%)**	230(71.4)	92(28.6)	322	
**Gender**				0.276
Male(n,%)	45(39.13)	15(32.60)	60	
Female(n,%)	70(60.87)	31(67.40)	101	
Age(year)^*^	62.89 ± 10.43	49.28 ± 10.96		0.000
Affected side^*^				0.000
Left(n,%)	38(33.04)			
Right(n,%)	77(66.96)			

^*^P 0.01

### Magnetic resonance imaging

MRI examinations were performed by a Philips Achieva 3.0T dual-source MRI scanner (Philips Healthcare, Amsterdam, The Netherlands). Balanced turbo field echo (BTFE) and enhanced T1 high-resolution isotropic volume excitation (ETHRIVE) sequences were obtained using a fast field echo (FFE) pulse sequence with repetition time (TR) = 25ms, echo time (TE) = 4.1ms, flip angle (FA) = 30°, acquisition matrix = 256 × 256, and slice thickness = 1.0mm. NVC was defined on the analysis of imaging with no perceptible CSF signal intervening between the vascular structure and the cisternal segment of the trigeminal nerve.

### Image processing

The dcm2niigui (http://people.cas.sc.edu/rorden/mricron/stats.html) was used to convert the original raw digital imaging and communications (DICOM) in medicine data of head MRA ETHRIVE and BTFE sequences into nifti (Neuroimaging Informatics Technology Initiative) format. Images were registered and fused using Co-register (Estimate & Re-slice) of the SPM12 (http://www.fil.ion.ucl.ac.uk/spm/software/spm12/) and opened by the 3D Slicer software (v4.11, https://www.slicer.org/) [14].

**Fig. 1:**
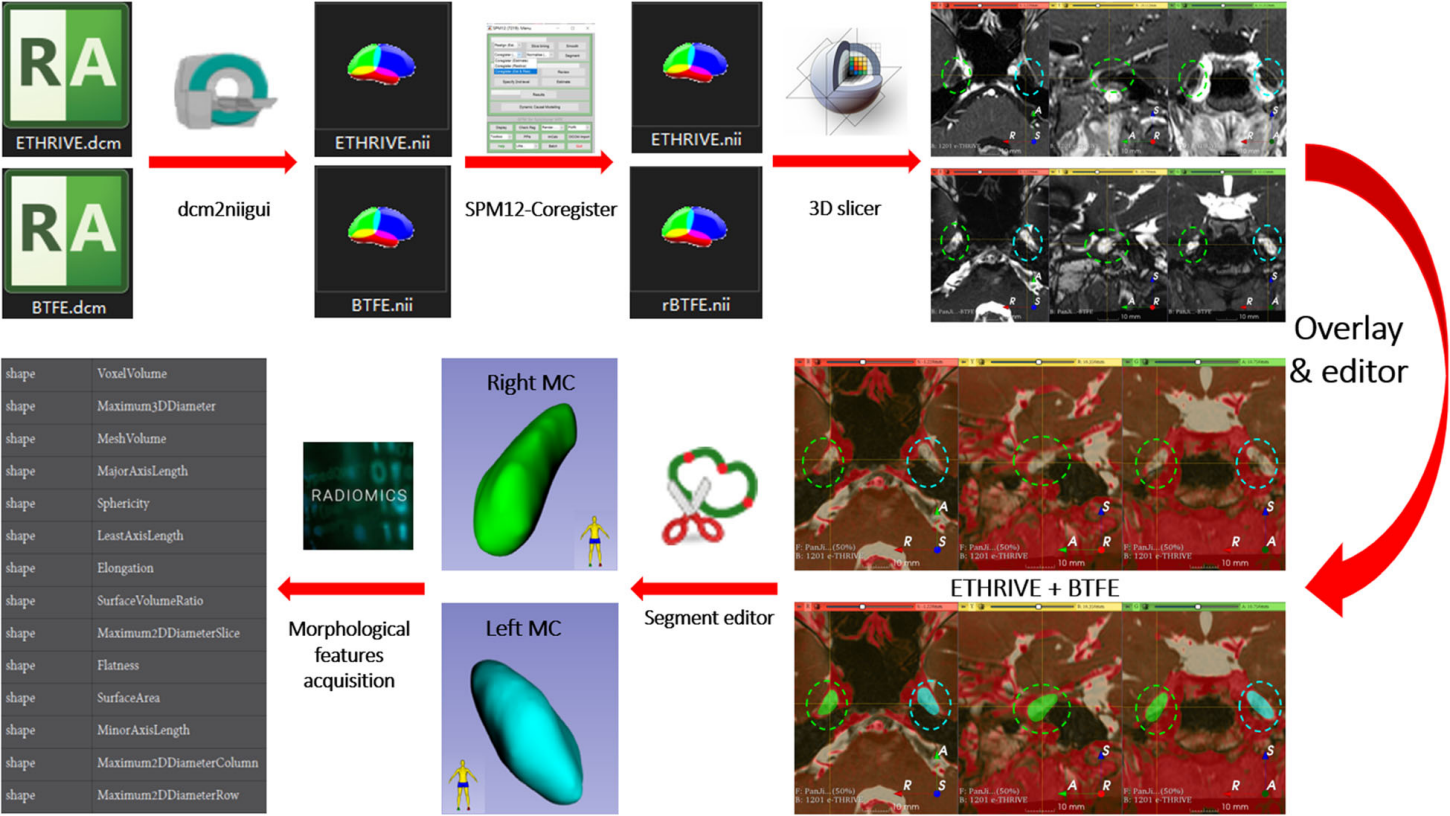
Morphological data acquisition flowchart

The segment-editor function of the 3D Slicer software was used to model and correct the bilateral MC in BTFE sequence through axial, coronal, and sagittal planes, and then registered ETHRIVE sequence was opened to overlap the BTFE sequence. At last, we observed the MC wall with ETHRIVE sequence, determined the edge of the MC from the axial, coronal, and sagittal planes, and recalibrated the edge of the MC again. (Fig. 1) All MRI studies and the manual plotting process were reviewed and analyzed by an experienced neuroradiologist and a neurosurgeon, who were blinded to the diagnosis and the laterality of the headache. When discrepancies existed, the two assessors reached a consensus for statistical analysis.

### Morphological features acquisition

Through the radiomics (revision:3b2c531) plug-in of the 3D Slicer software, we analyzed bilateral 322 MCs and got 13 morphological features of shape [15] including:

1. Voxel volume (using the following formula:

$${V}_{voxel}=\sum _{k=1}^{{N}_{v}}{V}_{k}$$

where the volume of the MC V_voxel_ is multiplying the number of voxels in the region of interest (ROI) by the volume of a single voxel V_k_.),

2. Surface area (using the following formula:

1 $${A}_{i}=\frac{1}{2} \left\arrowvert {{a}_{i}b}_{i }\times {{a}_{i}c}_{i } \right\arrowvert $$

2 $$A=\sum _{i=1}^{{N}_{f}}{A}_{i}$$

where a_i_b_i_ and a_i_c_i_ are edges of the i^th^ triangle in the mesh, formed by vertices a_i_, b_i_ and c_i_. To calculate the surface area, first the surface area A_i_ of each triangle in the mesh is calculated (1). The total surface area is then obtained by taking the sum of all calculated subareas (2).),

3. Maximum 3D diameter (is defined as the largest pairwise Euclidean distance between ROI surface mesh vertices. Also known as Feret diameter.),
4. Maximum 2D diameter slice (is defined as the largest pairwise Euclidean distance between ROI surface mesh vertices in the row-column (generally the axial) plane.),
5. Maximum 2D diameter column (is defined as the largest pairwise Euclidean distance between ROI surface mesh vertices in the row-slice (usually the coronal) plane.),
6. Maximum 2D diameter row (is defined as the largest pairwise Euclidean distance between ROI surface mesh vertices in the column-slice (usually the sagittal) plane.),
7. Minor axis length (using the following formula:

least axis=$$4\sqrt{{{\uplambda }}_{least}}$$

This feature yield the smallest axis length of the MC-enclosing ellipsoid and is calculated using the largest principal component λ_least_. In case of a 2D segmentation, this value will be 0.),

8. Major axis length (using the following formula:

major axis=$$4\sqrt{{\uplambda}\text{major}}$$

This feature yield the largest axis length of the ROI-enclosing ellipsoid and is calculated using the largest principal component λ_major_.),

9. Least axis length (using the following formula:

least axis=$$4\sqrt{{{\uplambda }}_{least}}$$

This feature yield the smallest axis length of the ROI-enclosing ellipsoid and is calculated using the largest principal component λ_least_. In case of a 2D segmentation, this value will be 0.),

10. Flatness (using the following formula:

Flatness=$$\sqrt{\frac{{{\uplambda }}_{least}}{{{\uplambda }}_{major}}}$$

Flatness shows the relationship between the largest and smallest principal components in the MC shape. Here, λ_major_ and λ_least_ are the lengths of the largest and smallest principal component axes. The values range between 1 (non-flat, sphere-like) and 0 (a flat object, or single-slice segmentation).

The model of the MC was independently performed by two neurosurgeons who were proficient in using the 3D Slicer software, and the morphological data were averaged.

### Statistical analysis

Chi-test were performed to assess differences in gender; Independent sample T test were performed to assess differences in age; Binomial test was used to test the left and right sides of PTN; Linear regression analysis were used to assess differences in morphological features of the MC (voxel volume, surface area, maximum 3D diameter, maximum 2D diameter slice, maximum 2D diameter column, maximum 2D diameter row, minor axis length, major axis length, least axis length, flatness); Paired sample T test were used to assess differences in morphological features of the bilateral MC of two groups respectively; and Kendall correlation analysis were used to assess correlation in the morphological features of the MC in PTN and HC groups using IBM SPSS Version 22.0 (IBM Corp., Armonk, New York, USA). *P* < 0.05 was considered statistically significant.

## Results

### Differences of demographic characteristics and morphological features of the MC between PTN and HC groups

The demographic characteristics of PTN and HC groups and the 10 morphological features of 322 MCs were analyzed. The right side incidence rate in the PTN group was higher than the left side incidence (66.96 % vs. 33.04 %, *P* < 0.01) (Table 1).

**Fig. 2:**
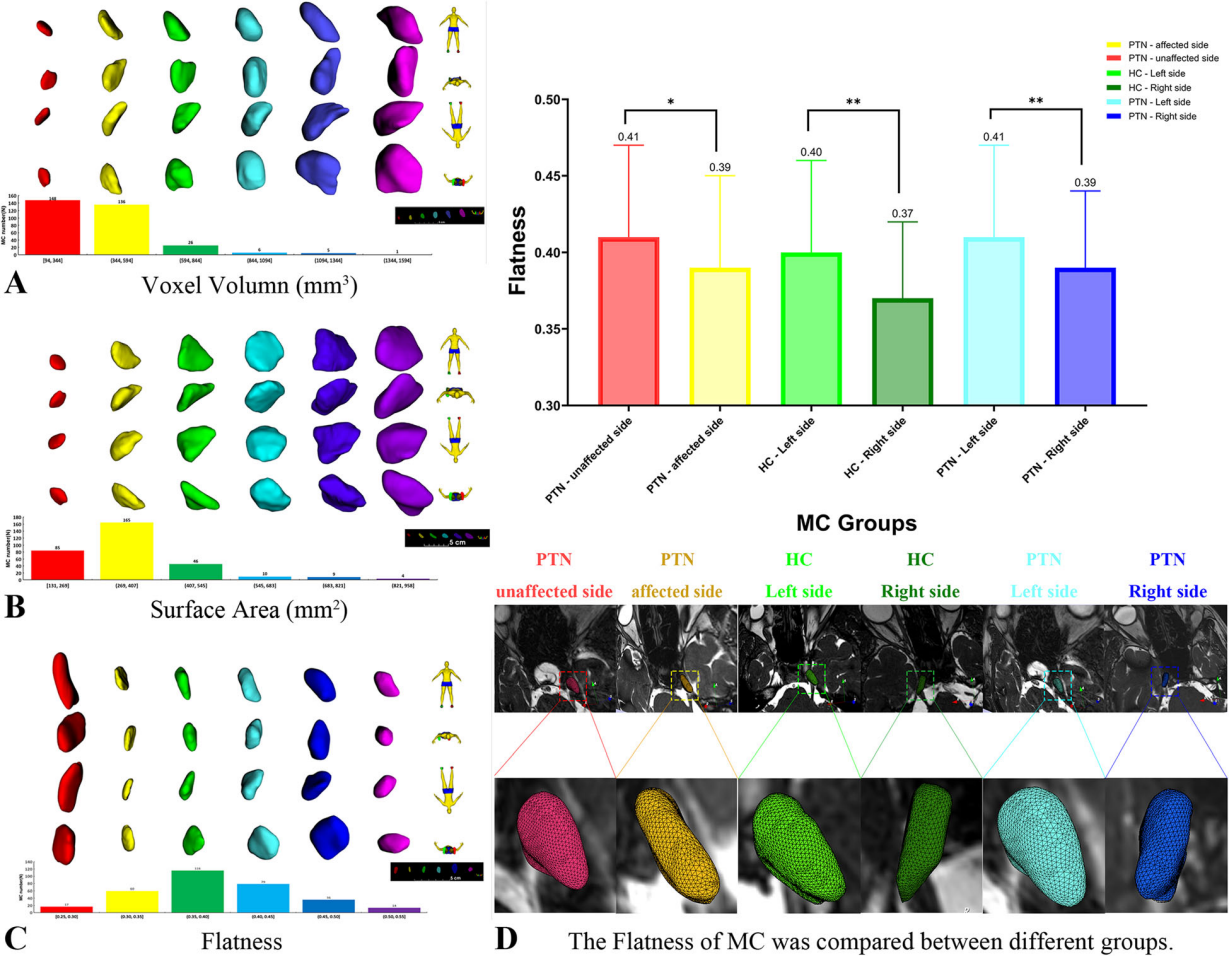
Morphology of MC with different morphological characteristics. (**A**) The red MC’s voxel volume is 94mm^3^, the yellow is 347mm^3^, the green is 601mm^3^, the light blue is 904mm^3^, the navy blue is 1136mm^3^ and the purple is 1589mm^3^.(**B**) The red MC’s surface area is 131mm^2^, the yellow is 335mm^2^, the green is 482mm^2^, the light blue is 569mm^2^, the navy blue is 784mm^2^ and the purple is 853mm^2^. (**C**) The red MC’s flatness is 0.25, the yellow is 0.32, the green is 0.37, the light blue is 0.42, the navy blue is 0.47 and the purple is 0.54.(**D**) The flatness of MC was compared between different groups.**P < 0.01; *P < 0.05

The voxel volume of the MC ranged 94.23 ~ 1589.90mm^3^, the surface area ranged 131.16 ~ 958.39mm^2^ (Fig. 2), the maximum 3D diameter ranged 8.42 ~ 19.70mm, the maximum 2D diameter slice ranged 7.28 ~ 16.88mm, the maximum 2D diameter column ranged 7.37 ~ 19.10mm, the maximum 2D diameter row ranged 8.03-19.64mm, the minor axis length ranged 6.50 ~ 12.46mm, the major axis length ranged 7.32 ~ 18.46mm, the least axis length ranged 2.84 ~ 5.98mm, there was no significant difference between PTN and HC groups (Table 2).

**Table 2:** Morphological analysis of MC

	HC($\stackrel{-}{x}$±s)	PTN($\stackrel{-}{x}$±s)		PTN($\stackrel{-}{x}$±s)			PTN($\stackrel{-}{x}$±s)			HC($\stackrel{-}{x}$±s)		
	Both sides	Affected side	*P*	Affected side	Unaffected side	*P*	Left side	Right side	*P*	Left side	Right side	*P*
MC number N(%)	92(44.4)	115(55.5)		115(50)	115(50)		115(50)	115(50)		46(50)	46(50)	
Voxel Volume (mm^3^)	369.88 ± 146.30	402.36 ± 213.12	0.214	402.36 ± 213.12	400.19 ± 219.07	0.815	397.14 ± 219.88	405.4 ± 212.21	0.163	367.72 ± 141.08	372.05 ± 152.88	0.810
Surface Area (mm^2^)	337.02 ± 97.25	361.95 ± 144.07	0.153	361.95 ± 144.07	355.28 ± 143.13	0.639	350.79 ± 144.72	366.44 ± 140.09	0.003^#^	330.96 ± 90.62	343.09 ± 104.1	0.101
Maximum 3D Diameter (mm)	0.96 ± 0.13	0.96 ± 0.17	0.889	14.19 ± 2.59	13.97 ± 2.47	0.526	13.79 ± 2.50	14.37 ± 2.53	0.000^#^	13.64 ± 2.09	14.09 ± 2.19	0.029
Maximum 2D Diameter Slice (mm)	13.86 ± 2.14	14.19 ± 2.59	0.339	12.97 ± 2.32	12.89 ± 2.37	0.789	12.68 ± 2.44	13.18 ± 2.21	0.003^#^	12.63 ± 2.07	12.94 ± 1.94	0.174
Maximum 2D Diameter Column (mm)	12.78 ± 2.00	12.97 ± 2.32	0.537	11.92 ± 2.62	11.53 ± 2.43	0.245	11.52 ± 2.49	11.93 ± 2.58	0.002^#^	11.29 ± 1.98	11.71 ± 2.28	0.039^*^
Maximum 2D Diameter Row (mm)	11.50 ± 2.13	11.92 ± 2.62	0.219	12.83 ± 2.48	12.65 ± 2.31	0.563	12.53 ± 2.38	12.95 ± 2.4	0.018^*^	12.38 ± 2.04	12.32 ± 2.13	0.725
Minor Axis Length (mm)	12.35 ± 2.07	12.83 ± 2.48	0.139	9.77 ± 1.94	9.58 ± 1.90	0.473	9.52 ± 1.94	9.83 ± 1.90	0.004^#^	9.34 ± 1.43	9.65 ± 1.65	0.067
Major Axis Length (mm)	9.49 ± 1.54	9.77 ± 1.94	0.276	12.17 ± 2.22	11.90 ± 2.02	0.346	11.81 ± 2.09	12.26 ± 2.14	0.001^#^	11.87 ± 1.94	12.17 ± 1.99	0.054
Least Axis Length (mm)	12.02 ± 1.96	12.17 ± 2.22	0.618	4.70 ± 0.80	4.80 ± 0.88	0.367	4.82 ± 0.88	4.68 ± 0.80	0.024^*^	4.67 ± 0.74	4.47 ± 0.61	0.011^*^
Flatness	4.57 ± 0.68	4.70 ± 0.80	0.219	0.39 ± 0.06	0.41 ± 0.06	0.040^*^	0.41 ± 0.06	0.39 ± 0.05	0.000^#^	0.40 ± 0.06	0.37 ± 0.05	0.002^#^

^*^P ˂0.05^#^*P* ˂0.01

By analyzing the flatness feature of the MC, we found that the flatness of MC ranged from 0.25 ~ 0.54, the affected side of the PTN was lower than that of the unaffected side (*P* < 0.05), the right MC of HCs and patients with PTN was lower than left MC (*P* < 0.01). (Fig. 2)(Table 2).

### Differences in morphological features of bilateral MC in PTN and HC groups

By analysing the morphological features of MC in the HC, we found that in some features, the left side and right side were significantly different. These differences included the maximum 3D diameter (*P* < 0.05), maximum 2D diameter column (*P* < 0.05), least axis length (*P* < 0.05), and flatness (*P* < 0.01) (Table 2).

As for the morphological characteristics of MC in PTN, we also found that there were significant differences between the left side and the right side in some features. These differences included the surface area (*P* < 0.01), maximum 3D diameter (*P* < 0.01), maximum 2D diameter slice (*P* < 0.01), maximum 2D diameter column (*P* < 0.01), maximum 2D diameter row (*P* < 0.05), minor axis length (*P* < 0.01), major axis length (*P* < 0.01), least axis length (*P* < 0.05), and flatness (*P* < 0.01) (Table 2).

### Correlation analysis of MC Flatness features in PTN and HC groups

The flatness of HC was positively correlated with the least axis length (*r* = 0.325, *P* < 0.01) and negatively correlated with the major axis length (*r*=-0.263, *P* < 0.01), maximum 3D diameter (*r* = -0.217, *P* < 0.01), maximum 2D diameter slice (*r* = -0.164, *P* < 0.05), maximum 2D diameter row (*r* = -0.169, *P* < 0.05). The most significant correlation was least axis length, followed by major axis length.

**Fig. 3:**
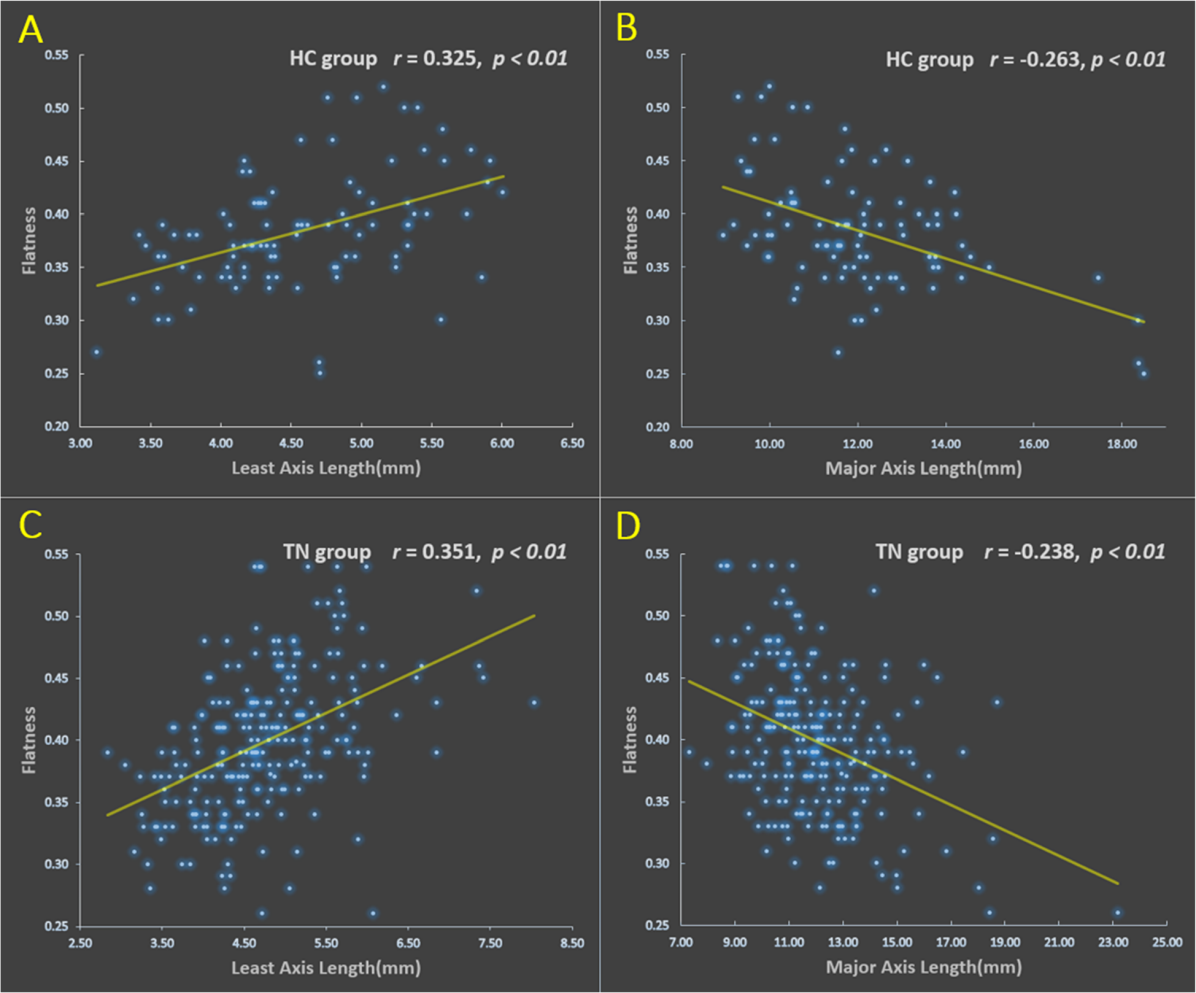
Scatter plot of correlation coefficient of flatness. (**A**) Scatter plots of the flatness positively correlated with the least axis length in the HC group (r = 0.325, *P* < 0.01). (**B**) Scatter plots of the flatness negatively correlated with the major axis length in the HC group (r = -0.263, *P* < 0.01). (**C**) Scatter plots of the flatness positively correlated with the least axis length in the TN group (r = 0.351, *P* < 0.01). (**D**) Scatter plots of the flatness negatively correlated with the major axis length in the TN group (r = -0.238, *P*< 0.01).

The flatness of PTN was positively correlated with the least axis length (*r* = 0.351, *P* < 0.01), major axis length (*r* =-0.238, *P* < 0.01), and negatively correlated with the maximum 3D diameter (*r* = -0.210, *P* < 0.01), maximum 2D diameter slice (*r* = -0.152, *P* < 0.01), maximum 2D diameter column (*r* = -0.109, *P* < 0.05) and maximum 2D diameter row (*r* = -0.147, *P* < 0.05). The most significant correlation was least axis length, followed by major axis length (Fig. 3).

### Differences in morphological features of the MC in PTN group without bilateral NVC

**Fig. 4:**
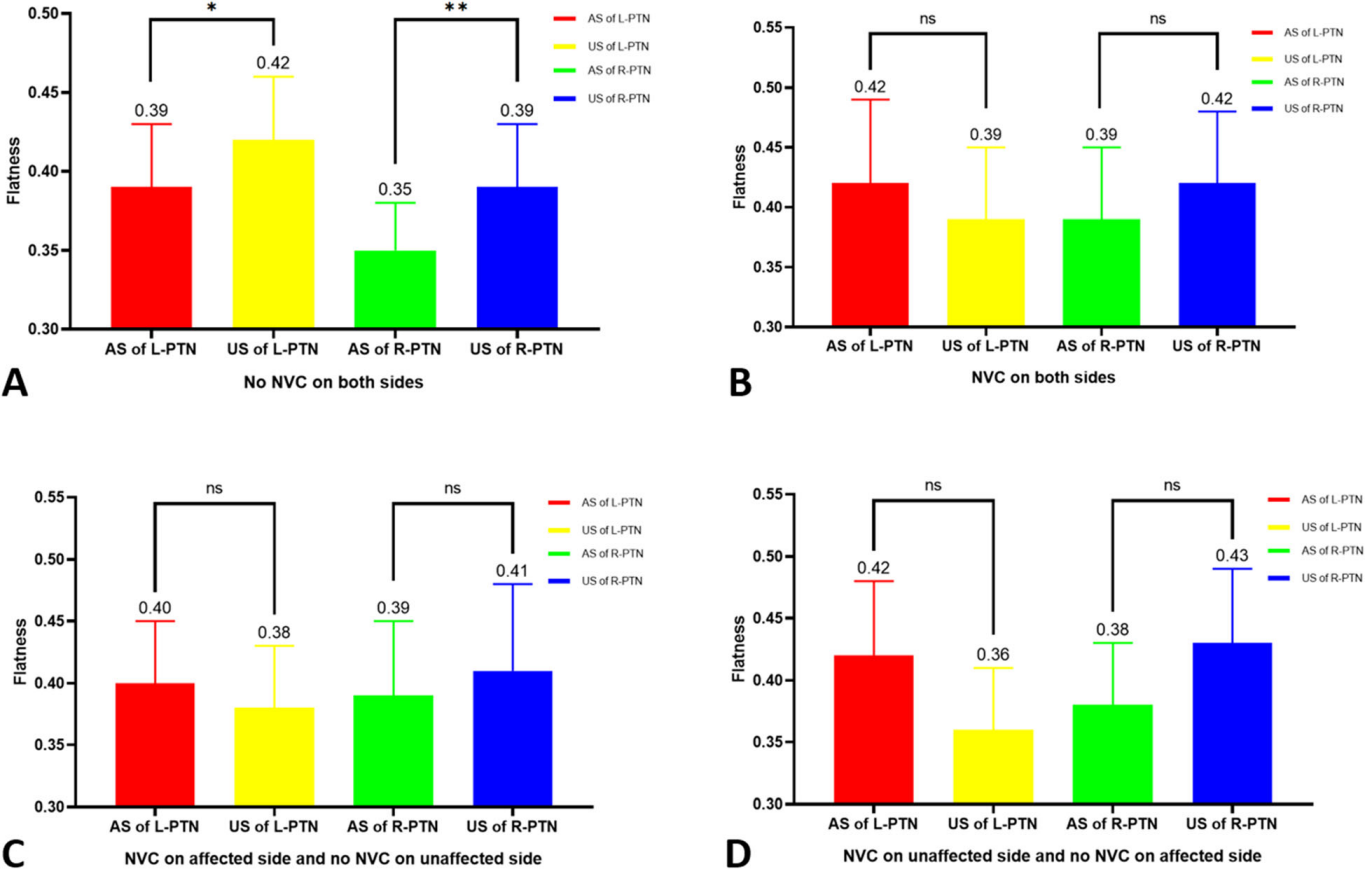
Comparative analysis of Flatness with bilateral MCs under different NVC conditions. (**A**) No NVC on both sides. The MC of the affected side of the left PTN without bilateral NVC was lower than that of the unaffected side (0.39 ± 0.04 vs 0.42 ± 0.04, *P* < 0.051), the MC of the affected side of the right PTN without bilateral NVC was lower than that of the unaffected side (0.35 ± 0.03 vs 0.39 ± 0.04, *P* < 0.05). (**B**) NVC on both sides. There was no significant difference between groups. (**C**) NVC on affected side and no NVC on unaffected side. There was no significant difference between groups. (**D**) NVC on unaffected side and no NVC on affected side. There was no significant difference between groups.*P < 0.051; ** P < 0.05; ns:*P *> 0.05; NVC: neurovascular contact;L: Left; R: Right; AS: Affected side; US: Unaffected side.

Table 3 summarizes the NVC of PTN group. There were 9 patients with left PTN and 9 patients with right PTN without bilateral NVC. The flatness of MC of the left PTN on the affected side was lower than that of the unaffected side (*P* < 0.051), the flatness of MC of right PTN on the affected side was lower than that of the unaffected side (*P* < 0.05). (Table 4)(Fig. 4) Other morphological features were no significant differences.

**Table 3:** Neurovascular contact (NVC) in PTN

		NVC			
	Affected side		Unaffected side		Total (n, %)
Left PTN (n = 38)	Yes		No		12 (31.6)
	No		Yes		4 (10.5)
	Yes		Yes		13 (34.2)
	No		No		9 (23.7)
Right PTN (n = 77)	Yes		No		34 (44.2)
	No		Yes		9 (11.7)
	Yes		Yes		25 (32.5)
	No		No		9 (11.7)

**Table 4:** Morphological analysis of MC in PTN group without bilateral NVC

	Left PTN			Right PTN		
	Affected side	Unaffected side	*P*	Affected side	Unaffected side	*P*
MC number N(%)	9(50)	9(50)		9(50)	9(50)	
Voxel Volume (mm^3^)	346.07 ± 133.75	377.69 ± 138.02	0.264	461.23 ± 325.48	571.21 ± 482.64	0.334
Surface Area (mm^2^)	321.01 ± 87.72	336.7 ± 84.62	0.389	398.94 ± 194.45	422.07 ± 219.35	0.609
Maximum 3D Diameter (mm)	13.39 ± 1.84	13.94 ± 1.75	0.247	14.93 ± 3.60	15.47 ± 3.35	0.469
Maximum 2D Diameter Slice (mm)	12.48 ± 2.28	12.82 ± 2.21	0.526	13.57 ± 2.83	14.22 ± 3.53	0.330
Maximum 2D Diameter Column (mm)	11.59 ± 1.61	11.43 ± 1.70	0.567	12.85 ± 3.39	12.51 ± 3.74	0.535
Maximum 2D Diameter Row (mm)	11.77 ± 1.87	12.25 ± 1.61	0.485	13.74 ± 3.77	13.76 ± 3.49	0.985
Minor Axis Length (mm)	9.43 ± 1.62	9.31 ± 1.56	0.701	10.59 ± 3.00	10.31 ± 3.00	0.630
Major Axis Length (mm)	11.67 ± 1.38	11.76 ± 1.28	0.815	12.85 ± 3.09	13.27 ± 2.97	0.457
Least Axis Length (mm)	4.51 ± 0.65	4.89 ± 0.71	0.090	4.42 ± 0.96	5.17 ± 1.49	0.052
Flatness	0.39 ± 0.04	0.42 ± 0.04	0.0509^#^	0.35 ± 0.03	0.39 ± 0.04	0.044^*^

^#^*P* ˂0.51 ;^*^P ˂0.05

## Discussion

The MC is an important anatomical part of the intracranial trigeminal nerve after the REZ. The MC is the dural space formed by the trigeminal nerve root, which is enclosed by the dural membrane of the posterior fossa in the middle fossa and crosses the rocky ridge; moreover, it contains the trigeminal ganglion and the trigeminal cistern [16]. Morphological changes in the MC are very important in the diagnosis [17–22] and treatment [23, 24] of intracranial diseases, such as radiofrequency thermocoagulation and PBC of PTN [25–29]. Ipsilateral hypoplastic MC and crowded MC have been suggested to produce symptoms consistent with TN [8, 9] At present, there is little research on the morphology of MC in patients with PTN and healthy people, and therefore a lack of statistical data comparing the MC morphology between these two groups.

In this study, we introduced the method of MC morphological features extraction based on radiomics. In the identification of MC anatomy by MRI, most studies [8, 17–19] have only used one of the T1 or T2 sequences. T1 enhanced sequence is helpful to determine the wall of the MC [30], but lacks the sensitivity of cerebrospinal fluid and nerves in the MC. T2 sequence can distinguish the MC content sensitively but cannot display the wall of the MC clear enough. We used the fusion of ETHRIVE and BTFE sequences of 3.0T MRI to model the MC, which can clearly show not only the structure in the MC but also the MC wall.

Radiomics is an automatic algorithm to quantify the phenotypic characteristics of medical imaging, which can be used after installing PyRadiomics plug-in through the 3D Slicer software [12] and is widely used in medical science research [31–34]. In this study, we obtained morphological features of the MC by radiomics, which can be divided into two categories. One is the feature that describes the size of the MC, including voxel volume, surface area, maximum 3D diameter, maximum 2D diameter slice, maximum 2D diameter column, maximum 2D diameter row, minor axis length, major axis length, and least axis length. The other feature describes the shape features of the MC, including sphericity and flatness.

The morphological results (voxel volume and surface area) of the MC in this study were significantly higher than those of an autopsy [35], which is due to the loss of MC cerebrospinal fluid in the cadaver head as well as the fixation of the corpse.

The precise acquisition of MC morphological data through the radiomics method is beneficial for the preparation of PBC for PTN. PBC requires the puncture of the MC and filling up of the balloon to compress the semilunar ganglion nerve [36]. The accurate evaluation of the MC volume before PBC can not only provide a reference for the balloon filling volume but also prepare appropriate balloon volume for some special MC volumes. (The maximum volume of MC in this study was 1589.90 mm^3^.)

The shape analysis of the MC is difficult to quantify and is generally described as an ellipse or open-ended three-fingered glove [37]. By contrast, the flatness was obtained in this study using radiomics and was accurately quantified and analyzed.

In the HC group, the maximum 3D diameter and maximum 2D diameter column of the right MC were greater than those of the left MC (*P* < 0.05), whereas the least axis length and flatness were less than those of the left MC (*P* < 0.05). In the PTN group, the surface area, maximum 3D diameter, maximum 2D diameter slice, maximum 2D diameter column, maximum 2D diameter row, minor axis length, and major axis length of the right MC were greater than those of the left MC (*P* < 0.05), while the least axis length and flatness were less than those of the left MC (*P* < 0.05), suggesting that the shape of the bilateral MC was asymmetric. The MC on the right side was flatter and larger than that on the left side, especially in the PTN group. To our knowledge, the bilateral asymmetry of the MC has been rarely described in the literature among healthy people and patients with PTN. There is an asymmetry between the two hemispheres of the human brain, including the cortical projection area associated with the trigeminal nerve, such as the posterior central gyrus, cingulate gyrus, and paracentral gyrus [38–40]. Whether the asymmetry of the MC shape is related to the volume or thickness of the cerebral cortex deserves further study.

The complicated pathogenesis of PTN has always been a focus and is unclear, to a large extent, in medical knowledge. Although the most common pathogenic mechanism is NVC in the REZ [41], asymptomatic vessel-nerve contact is present in 92 % of patients, and there is no NVC in 3.1–17 % of PTN patients [4]. Therefore, this theory does not apply to all the cases [42]. A study [43] reported 6 cases having whiplash-associated disorder with specific symptoms of trigeminal neuralgia-like pain, and some cases had hyperesthesia. Therefore, it may be classified as painful post-traumatic trigeminal neuropathy rather than PTN based on the third edition of the International Classification of Headache Disorders [13].

By analyzing the flatness features of the MC, we found that the affected side of the PTN was lower than that of the unaffected side (*P <* 0.05), and that the affected MC side of the left PTN without bilateral NVC was lower than that of the unaffected side (*P* < 0.051). Moreover, the affected MC side of the right PTN without bilateral NVC was lower than that of the unaffected side (*P* < 0.05). These results suggest that flatness was still associated with PTN even after NVC exclusion. Further analysis found that the right MC of HCs and patients with PTN was lower than that of the left MC (*P* < 0.01) and that the prevalence of PTN on the right side was higher than that on the left side (*P* < 0.01), which was consistent with the literature [2, 44]. Our current study suggests that this finding is more than a mere chance association, and that the flatness of the MC may be associated with PTN, which is likely to explain the high prevalence of the right PTN.

Based on the results of this study, we found that the right MC is larger and flatter than the left MC, which may lead to increased density or compression of the trigeminal nerve at the right MC. This compression of the trigeminal nerve can lead to PTN. Ipsilateral MC dysplasia may produce symptoms consistent with TN [8] and crowded MC may also lead to TN [9] Therefore, we believe that the flatness of the MC may be a cause of PTN.

The limitation of this study is that the *P*-value of the MC flatness results on the affected MC side of the left PTN without bilateral NVC was 0.0509. Despite being very close, it did not reach the statistical level of significance (*P* < 0.05), which was related to the insufficient sample size of this study. In addition, the mean ages of the PTN and HC groups had a significant difference, which was related to the age of onset of TN and the small number of individuals in the HC group. Further research will expand the sample size of the HC group and select the corresponding ages for comparison. We also only analyzed the morphological characteristics of the MC without further analysis of the trigeminal nerve in the MC. The next step is to increase the number of samples and analyze the trigeminal nerve components in the MC to explore the etiology of PTN.

## Conclusions

By providing a method to analyze the morphology of the MC, we found that there is an asymmetry in the morphology of bilateral MC in the PTN and HC groups. It can be inferred that the flatness of the MC may be a cause of PTN.

## Data Availability

The data that support the findings of this study are available from the corresponding author, upon reasonable request.

## Abbreviations

HC: healthy controls
MRI: magnetic resonance imaging
MC: Meckel cave
NVC: neurovascular contact
PBC: percutaneous balloon compression
PTN: primary trigeminal neuralgia
REZ: root entry zone
TN: trigeminal neuralgia
BTFE: balanced turbo field echo
ETHRIVE: enhanced T1 high-resolution isotropic volume excitation
ROI: Region of interest
